# Physicochemical, Sensorial and Microbiological Characterization of PoroCheese, an Artisanal Mexican Cheese Made from Raw Milk †

**DOI:** 10.3390/foods8100509

**Published:** 2019-10-17

**Authors:** Citlalli Celeste González Ariceaga, Muhammad Inam Afzal, Muhammad Umer, Shabbar Abbas, Haroon Ahmad, Muhammad Sajjad, Fahed Parvaiz, Kaleem Imdad, Muhammad Imran, Abid Aslam Maan, Muhammad Kashif Iqbal Khan, Azmat Ullah, Arturo Hernández-Montes, Eleazar Aguirre-Mandujano, Abraham Villegas de Gante, Muriel Jacquot, Catherine Cailliez-Grimal

**Affiliations:** 1 Biomolecular Engineering Laboratory, University of Lorraine, F-54518 Nancy, France; muriel.jacquot@univ-lorraine.fr; 2 Departamento de Zootecnia, Universidad Autónoma Chapingo, Carretera México-Texcoco km 38.5, Texcoco CP 56230, Mexico; hermora@prodigy.net.mx (A.H.-M.); eleagman@yahoo.com (E.A.-M.); abecamus@gmail.com (A.V.d.G.); 3 Department of Biosciences, COMSATS University Islamabad, Park road, Tarlaikalan, Islamabad 45550, Pakistan; umer.imperial@gmail.com (M.U.); shabbar.abbas@comsats.edu.pk (S.A.); haroonahmad12@yahoo.com (H.A.); msajjadpbg@gmail.com (M.S.); fahed.pervaiz@comsats.edu.pk (F.P.); kaleem.imdad@comsats.edu.pk (K.I.); 4 University Institute of Diet and Nutritional Sciences, Faculty of Allied Health Sciences, The University of Lahore, Lahore 54000, Pakistan; mic_1661@yahoo.com; 5 Department of Food Engineering, University of Agriculture, Faisalabad 38000, Pakistan; abid.maan@uaf.edu.pk (A.A.M.); kashif.khan@uaf.edu.pk (M.K.I.K.); 6 Department of Food Science and Human Nutrition, University of Veterinary and Animal Sciences, Out Fall Road, Civil Lines, Lahore 54000, Pakistan; azmat.khan@uvas.edu.pk; 7 Stress Immunity Pathogens Laboratory, University of Lorraine, EA7300 Nancy, France

**Keywords:** artisanal cheese, physicochemical characteristics, sensory analysis

## Abstract

Poro cheese is a regional product originally from the area of Los Rios, Tabasco in Mexico. In the context of preserving the heritage of Poro cheese and protecting the specific characteristics that define its typicity through an origin designation, the present study was conducted to establish a general profile of Poro cheese by characterizing their physicochemical, textural, rheological, sensorial and microbiological characteristics. Differences in moisture, proteins, fats, NaCl, titrable acidity, pH, color texture and rheology amongst cheese factories were observed and ranges were established. Fifteen descriptors were generated to provide a descriptive analysis, eight of which were significantly different amongst the factories with no differences in the global acceptability of cheese. The favorite cheese had the highest scores for aroma attributes. Conventional and molecular methods were used to identify the main microorganisms, for which *Lactobacillus plantarum, L. fermentum, L. farciminis* and *L. rhamnosus* were the main microorganisms found in Porocheese. The obtained data constituted the parameters for characterizing Poro cheese, which will strongly help to support its origin appellation request process.

## 1. Introduction

**Figure 1:**
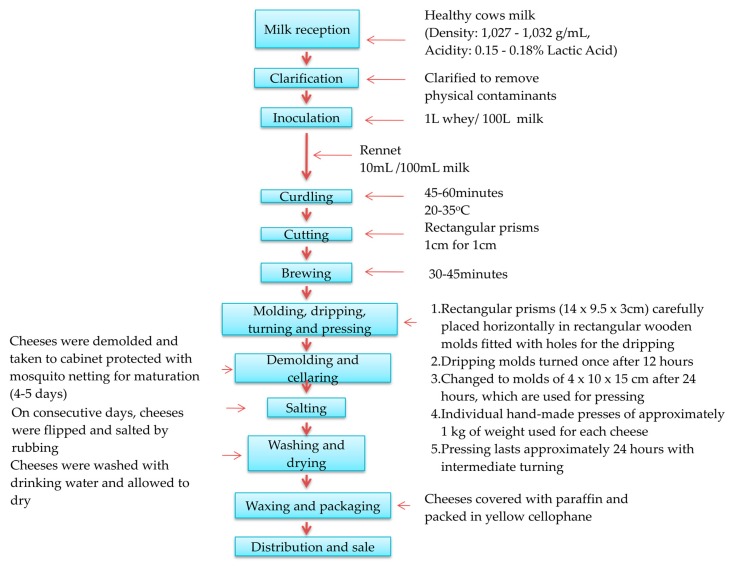
Flowchart for Poro cheese making process.

Poro cheese is a handmade product originally from the region of Los Rios, Tabasco in Mexico [1]. The seven-day Poro cheese manufacturing process is characterized by several stages: milk reception and filtering, temperature verification, acidification and coagulation by whey addition from the previous day, rennin addition, curd cutting, whey draining, molding, draining, pressing, salting, rest, airing, coating, wrapping for sale and distribution (Figure 1). Poro cheese is usually made from raw milk of Cebuino cattle and its crosses with European breeds and has a short shelf life. It is produced in pieces ranging from 250 to 400 g and its maturation is light. One of its distinctive features is the paraffin layer that covers each piece during packaging. Raw cow milk and natural ingredients are allowed in cheese making process of Poro cheese, such as liquid rennet (rennin) and salt. The product is presented as small rectangular prisms of the following dimensions: 14 cm (length) by 9.5 cm (width) and 3 cm (height), approximately. The pieces covered with white paraffin are usually wrapped in a thin yellow-orange cellophane film. Its type of paste is semi-hard with low humidity, small longitudinal “eyes” (mechanical), formed by the layered arrangement of the curd during molding. The paste experiences a slight ripening during the cheese making (which takes seven days). It has a very low pH (4.34 on average) in relation to most Mexican cheeses and compared to other world famous cheeses which come from an enzymatic curdling of milk. Also, its water activity (aw) is relatively low (0.94 average), which is consistent with its moisture content and its salt content, relatively high (3.2%, average) [1,2]. The pH, aw, and salt concentration values, together with the absence of coliform microflora in two-week cheeses, allows to infer that even when they are made with tropical raw milk, they could cover the safety requirement for consumers. 

There is no official data available on the production and consumption of Poro cheese in specific. However, the total production of artisanal cheese in Mexico is twice that officially reported and it is estimated at 410 thousand tons [1,3]. 

When talking about a product whose history, tradition and typicity are anchored with a place and a society, surrounded by a know-how that is transmitted throughout time, the appellation acquires a patrimonial dimension, which gives the products an authentic prestige. Ultimately, this forms part of a common patrimony, which society will strive to protect. Products with a strong identity have a better market positioning because buyers can identify their quality attributes, which are governed by the know-how of the manufacturer [4]. This situation represents a great challenge for artisanal Mexican and world cheese producers. Only 13 prestigious product designations for Mexican products are known (InstitutoMexicano de la Propiedad Industrial, 2012). On worldwide scale, several artisanal cheeses have received extensive characterization to establish unique attributes of cheese varieties, enabling the preservations of these products and their attributes. Examples of those are Sepet and Enzine cheese from Turkey, Majorero and Murcia al Vino cheeses from Spain and Añejo, and Ranchero cheese from Mexico [4,5,6,7,8,9].

Previously, we proposed a novel alternative method for the incorporation of volatile compounds in Poro cheese using direct encapsulation of *C. maltaromaticum* LMA 28 for improving sensory attributes [10]. In the context of preserving the heritage of Poro cheese and protecting the specific characteristics, which define its typicity through a collective brand or an origin designation, the objective of this work was to establish a general profile of Poro cheese by characterizing their physicochemical, microbiological, rheological and sensory properties. 

## 2. Materials and Methods 

### 2.1. Sampling

Cheese samples were collected from five different artisanal cheese factories in Balancán, Tabasco, Mexico. Factories were chosen by their location, prestige and product availability. Cheese units were taken in triplicates at the end of their seven days elaboration process; they were stored in a cool container at 4°C and transported to the laboratory. All the analyses were performed on cheese samples from five cheese factories (QP1, QP2, QP3, QP4 and QP5); microbiological characterization was carried out on two potentially important cheese samples from two cheese factories (QP1 and QP3). Factory names were coded for confidentiality reasons.

### 2.2. Physicochemical and Texture Analysis

Fat content by Gerber-Van Gulik [11], moisture [12] (method 926.08, AOAC 1995), protein [12] (935 method, AOAC 1995), ash [12] (method 935.42, AOAC 1995) and NaCl [12] (method 935.43, AOAC 1995) were determined as well as pH with a potentiometer HI 9230 (Hanna Instruments, Limena, Italy). A textural profile analysis (TPA) was performed using a TA-Xt2i texture analyzer (Stable Micro Systems, Surrey, UK) and Texture Expert 7.15 H software (Stable Micro Systems, Surrey, UK) with a load cell of 5 kg. Measurements were performed in triplicate and hardness, adhesiveness, cohesiveness, springiness and chewiness were calculated from the double bite curves. 

### 2.3. Rheological Analysis

Dynamic oscillatory measurements were performed with a Physica MCR 301 (Physica Messtechnik, Stuttgart, Germany), with a parallel plate rough geometry, in which the rotating plate was 50 mm in diameter. Cylinders samples 50 mm in diameter were cut from the centre of the cheeses and they were wrapped in aluminum foil to prevent dehydration and tempered for 1h at room temperature [13]. The linear viscoelastic region of the cheeses being determined by amplitude sweeps, using deformation values of 10–3 to 100% and 1Hz frequency and 25 °C. Frequency sweeps were made from 0.01 to 100 Hz. The storage modulus (G’) and the loss modulus (G’’) of cheese samples were obtained from the equipment software by triplicate.

### 2.4. Color Measurement 

Internal cheese color was measured using a MiniScan 45/0 LAV (HunterLab, Hunter Associates Laboratory, EE.UU.). Hue and chroma were calculated using the CIElab scale with D65 as illuminant and an observer angle of 10°, yellowing index was also measured.

### 2.5. Sensorial Analysis

A trained panel of eight members performed a descriptive analysis for Poro cheese (Table 1). 

Judges were trained for 30 h. A 15 cm interval scale was used, where zero represented attribute absence and 15 a very high intensity. Cheese samples were randomly evaluated for each panelist in triplicate. Overall acceptability of Poro cheese was evaluated using a nine point hedonic scale by a panel of 101 consumers. On the other hand, cheese producers also evaluated the overall and specific attributes acceptability using a nine point hedonic scale. Evaluated specific attributes were the same used by the trained panel except for tactile hardness and tactile creaminess that were not used (Table 1).

### 2.6. Microbiological Enumeration

Ten grams of each cheese were aseptically taken and dissolved in 90 mL of sterile 2 % sodium citrate solution into a sterile stomach bag and homogenized for 2 min. Decimal dilutions were prepared in tryptone salt buffer and used for enumeration on agar plates. Culture media and incubation conditions are summarized in Table 2. After incubation, colony forming units (CFU) were counted and means and standard deviations were calculated.

### 2.7. Strains Isolation and Biochemical Identification

For the isolation, purification, characterization and identification of lactic acid bacteria (LAB) isolates, suspected colonies from different origin were randomly selected and subcultured. The identity of gram-positive, catalase-negative cocci and rods were determined by performing biochemical and physiological tests. Rod-shaped bacteria were tested for their ability to produce gas and to grow in Man, Rogosa and Sharpe (MRS) broth (Biokar, Beauvais, France) at 15 °C for seven days and 45 °C for two days. Cocci were tested for their ability to produce gas, to hydrolyse arginine and to grow in presence of 6.5% NaCl and 10% of bile in Elliker broth (Biokar, Beauvais, France) at 10 °C for ten days at 45 °C for 48 h. Carbohydrate fermentation patterns were determined using API 50 CH test strips (BioMérieux, Marcy l’Etoile, France) according to the manufacturer’s instructions.

### 2.8. DNA Extraction and PCR Amplification

Total genomic DNA was extracted from each isolate. Tubes containing 9 mL of Elliker or MRS broth were used to accommodate the isolated strains and incubated for 48 h at 30 °C or 37 °C. Two mL of each culture was used for DNA extraction using the method described previously [14]. DNA extraction from cheese samples were carried out according to the method described previously [15]. Bacterial DNA and cheese strains were treated with RNase and then 16S ribosomal genes were amplified using universal bacterial primers fD1 and rD1 [16]. The amplification program was: 95 °C for 5 min, followed by 35 cycles of 95 °C for 2 min, 45 °C by 30 s, 72 °C for 4 min, then 72 °C for 10 min, carried in a thermocycler (Bio-Rad Laboratoiries, Hercules, CA, USA). PCR products were stored at −20 °C until use.

### 2.9. Strains Identification and Time Temperature Gel Electrophoresis (TTGE) 

The PCR extracts from bacterial isolates were sequenced by GATC-biotech company (Konstanz, Germany). Partial sequences were compared to those in the GenBank database using the BLAST program. Cheese PCR products were subjected to a TTGE analysis using a Dcode universal mutation detection system (Bio-Rad Laboratories, Hercules, CA, USA) following the method described previously [14].

### 2.10. Statistical Analysis

A completely randomized design (DCA) was performed for physicochemical data, color, texture and rheology variables (*p* < 0.05). Averages were compared by the least significant difference test (LSD). For the descriptive analysis a randomized complete block design (RCB) with a split plot arrangement was used, while for the acceptability tests, a RCB was performed. The least significant difference method was used for comparison of means. Principal components analysis (PCA) was applied to obtain preference maps. The statistical calculations were performed using the statistical software SAS^®^ version 9.1 (SAS Institute, Inc., Cary, NC, USA), along with The Unscrambler^®^ version 9.2 (CAMO PROCESS AS, Oslo, Norway). 

## 3. Results and Discussion

### 3.1. Physicochemical Analysis

The physicochemical properties of Poro cheese (Table 3) indicated significant differences for all the parameters and those differences were probably related to the milk composition and cheese elaboration procedures, as previously suggested in Italian Fiore Sardo cheese [17]; in Argentinean Corrientes cheese [18] and in Mexican Añejo cheese [9]. In general, physicochemical properties of Porocheese ranged from 29.8 to 37.0% fat, 28.1 to 37.0% moisture, 24.5 to 39.4% protein, 2.0 to 3.9% NaCl, 0.12 to 0.28% Ca^2+^, 4.3 to 4.9 pH, and 0.930 to 0.955 for a_w_. These values were found consistent as reported for Añejo cheese [9] an artisanal semi-hard cheese from Mexico, but different to those reported for Ranchero cheese [4] another Mexican cheese considered as soft.

### 3.2. Texture Profile Analysis

Variations were observed in the mechanical properties of cheese among producers except for cohesiveness (Table 3). The TPA values of Poro cheese ranges from 11.92 N to 29.63 N for hardness, from −0.643 Ns to −0.0002 Ns for adhesiveness, from 0.38 to 0.47 for cohesiveness from 0.63 to 0.89 for springiness and from 4.08 to 9.97 for chewiness. Great variations among producers in TPA parameters were also reported in other artisanal cheeses such as Añejo and Ranchero Mexican cheese [4,9] and in Chihuahua cheese made with raw milk [19]. These important variations might be due to the variability in milk composition and the differences in the artisanal process between cheese makers. Similar TPA values were found in another Mexican artisan cheese (Ranchero cheese) except for the adhesiveness value, artisanal Ranchero cheese TPA values were 24.83 ± 12 N for hardness, −0.93 ± 0.4 Ns for adhesiveness, 0.4 ± 13 for cohesiveness, 0.76 ± 0.12 for springiness and 6.6 ± 2.03 N for chewiness [4]. The composition of cheese and milk influence the texture parameters of cheese. It is possible that the high hardness of the QP3 over the others is related to the fact that it was the least humid [20].

### 3.3. Rheological Analysis

**Figure 2:**
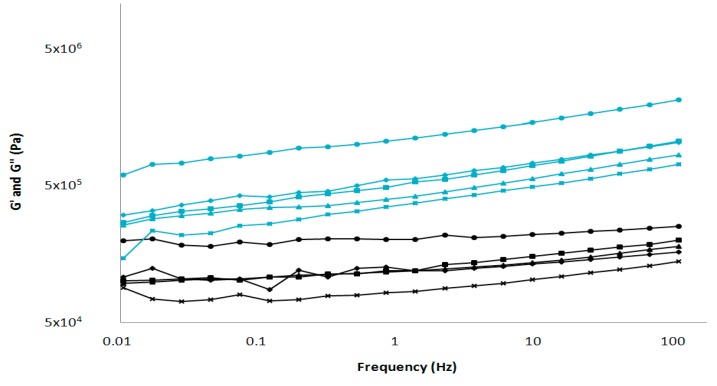
Average graph of storage modules (G’) and loss modules (G’’) for the five Poro cheese factories: (♦) QP1, (▲) QP2, (●) QP3, (✕) QP4 and (■) QP5.

The mechanical behavior of Poro cheese was determined by low amplitude oscillatory tests, dynamic measurements were carried out in the linear viscosity range, so cheese structure was preserved. Figure 2 showed the elastic modulus (G’) and the viscous modulus (G’’) for each cheese. In general all cheeses showed values of G’ greater than G’’ except for the first point on the QP1 graph. Furthermore, the values of G’ and G’’ increased with frequency increment exhibiting the viscoelastic nature of the material [21,22,23]. The results were similar to those described previously, where a significant increase in storage and loss modulus through ripening period was observed for Siahmazgi cheese [24], ewe’s milk cheese [25], Gaziantep cheese [26] and Valdeon cheese [27]. This increase in viscoelastic properties of might be related to fat reduction in processed cheese [28]. This is the typical behavior of a viscoelastic material with solid character and has been previously reported in cheese by several authors [29,30,31,32]. Values of G’ and G´´ for each cheese were mentioned in Table 3. There was a significant difference among the cheese factories, particularly for QP3 with the highest values and QP4 with the lowest values. Poro cheese G’ and G’’ ranged from 281.91 to 1081.14 and 67.4 to 177.92 kPa, respectively.The values of G’ and G’’ for Poro cheese were higher than those reported for other types of cheese. Chihuahua cheese, made from raw cow’s milk showed average values of 67.7 and 19.8 kPa for G’ and G’’ respectively [19]. Another study reported values ranging from 45 to 95 kPa (G’) and from 15 to 30 kPa (G”) according to the ripening period of Edam-type cheese [33].

### 3.4. Color Measurement

Color is a sensory attribute whose perception is highly variable and dependent on factors such as lighting, observation angle or observer. However, it is also a very important feature of cheese, because it is one of the first sensory attributes perceived before purchase or consume a product [9]. Table 3 showed the results of color measurements of Poro cheese. Cheeses samples present significant differences (*p* < 0.05). The values of Luminosity, Yellowness Index (YI), Chroma and Hueof Poro cheese ranged from 86.33 to 90.01, 31.89 to 40, 17.37 to 36.3 and 61.45 to 90.08, respectively. The composition of cows feeding has been associated with differences in color due to the higher content of carotenoids [34]. Although the difference in YI of the cheeses was not perceived by the trained judges, the cheese they perceived as less yellow, instrumentally was the brightest. Factors such as indigenous microflora, manufacturing process differences, ripening and storage conditions can also influence cheese final color and its perception [2]. 

### 3.5. Sensorial Analysis

The panel generated fifteen descriptive attributes to characterize the appearance, aroma, flavor and texture of Poro cheese. Table 1 showed those attributes, their definition and their reference values on the scale. The results of variance analysis and means comparison showed differences among cheese samples for eight of the fifteen attributes (Table 4). 

**Figure 3:**
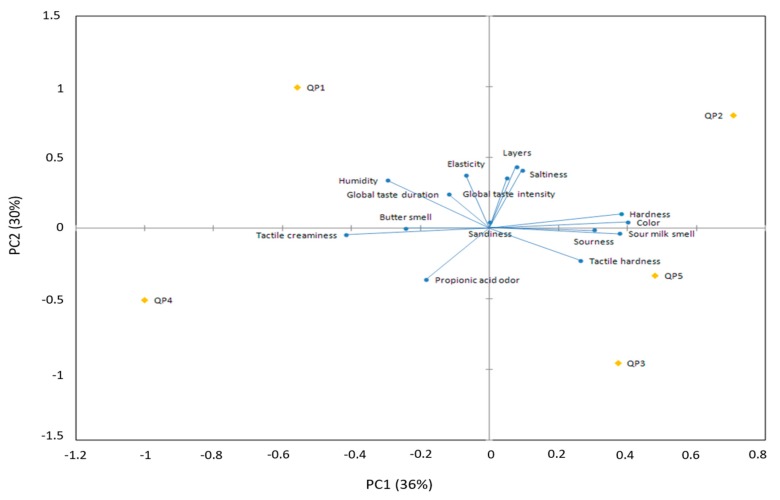
Principal components graphic for Poro cheese sensory attributes (loads) and cheeses (scores).

For color, the only difference was found in QP4, whose score was lower, which indicated the least yellow cheese. Instrumentally, this cheese was not the least yellow but cheeses QP2 and QP3 presented YI values above it. Regarding the layers space resolution, QP3 and QP4 had the lower space resolution. The production process of Poro cheese changed between cheese factories, like the operation affecting the layers (pressing), in QP3 dairy takes one day more than in the others. Additionally, QP4 was pressed using a Dutch type press, which caused QP3 and QP4 to have a more compact structure and therefore it was not easy to perceive the layers. No differences were found among the evaluated flavor attributes (sour milk aroma, butter aroma and propionic acid aroma). However, textural attributes (tactile hardness and tactile creaminess) differed between cheeses. QP3 was perceived as the hardest one. This data coincided with instrumental measurements. Cheeses perceived as creamier were QP4 and QP1. There was no significant difference for sandiness and elasticity evaluated in mouth. However, there were differences for humidity and hardness. The cheeses QP1, QP2 and QP4 were perceived as more humid, while QP1 and QP4 were less firm. There were no differences among cheese samples for saltiness but for sourness, QP2 and QP3 cheese samples were detected as more acid. The cheese global taste intensity of QP1, QP2 and QP3 was higher than the other, while the global taste permanence was lower for QP5. The PCA of descriptive data showed that the first two principal components (PCs) explained 66% of the variability of the cheeses descriptors. The first principal component (PC1) explained 36% of the data variability and correlate positively with the attributes of color, sour milk aroma, hardness and sourness and negatively with creaminess and humidity. The PC2 explained 30% of variance and was positively correlated with the attributes layers, elasticity, saltiness and global taste intensity. It was negatively correlated to propionic acid aroma (Figure 3). 

In a similar study of cream cheese, Brighenti et al. [35] found after a PCA that the first PC’s explained 93% of the variation. The PC1 was positively related to firmness, cohesiveness, spread and solubility and negatively related to stickiness, while PC2 explained elasticity and particle size. Another study [31] showed that the variability of the first two PC explained 96.1% of the variation. PC1 accounted for 55% and was positively correlated with the age of the cheese. PC2 accounted for 26.1% and was positively correlated with firmness, fracturability, elasticity and negatively with recovery [31].

No significant difference for overall acceptability was found among cheese samples evaluated by habitual consumers. When the producers evaluated the overall acceptability of cheeses, significant differences were found. In this case, cheese with the highest acceptability was QP4 and the one with the lowest was QP1. Besides the overall acceptability, the producers also rated the acceptability by attribute for each cheese. Producers found significant difference among cheese samples for nine of the fourteen attributes. 

**Figure 4:**
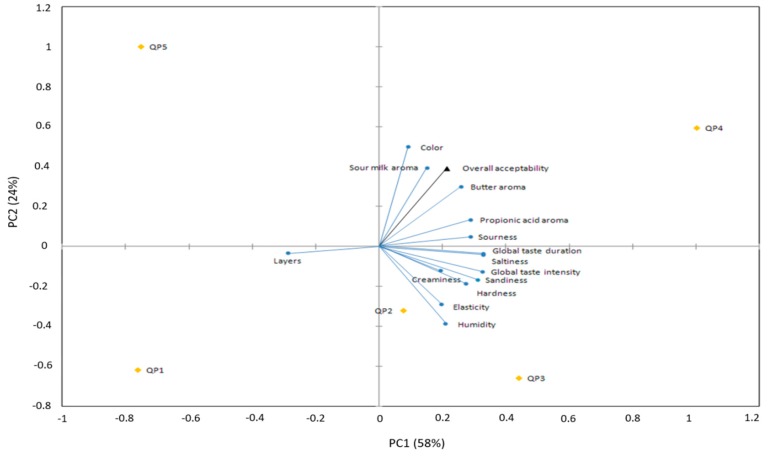
Poro cheese external preference map.

External preference map of Poro cheese is depicted in Figure 4. No finding acceptability difference among cheese samples could be explained by inexperience of consumers in comparison with producers. 

### 3.6. Microbiological Analysis

Among the five cheese factories, cheeses from QP1 and QP3 were chosen for microbiological analysis due to their significant difference concerning the physicochemical and sensorial data. The genera of LAB responsible for sensory attributes in milk and cheese generally comprised on *Lactococcus*, *Lactobacillus*, *Streptococcus*, *Carnobacterium* and *Enterococcus* [36,37,38,39,40,41,42,43]. 

**Figure 5:**
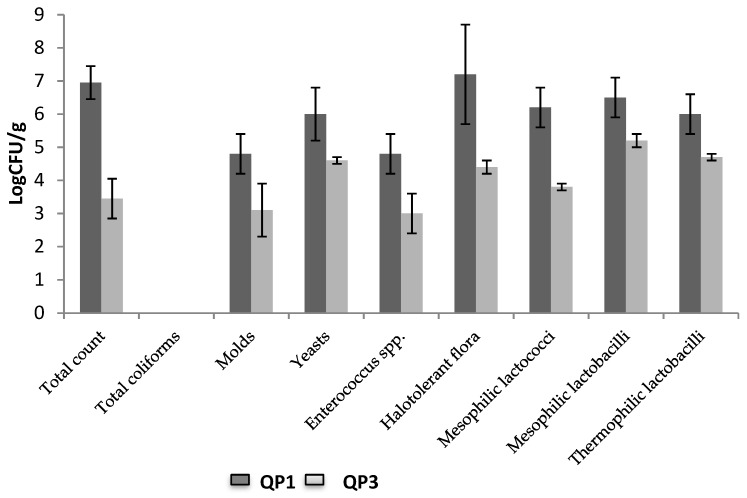
Average bacterial population in Poro cheese (Log CFU/g).

The microbial population present in Poro cheese was determined by conventional microbiological analyses using different culture media. Samples from the two different productions (QP3 and QP1) were used to obtain the average bacterial population (Figure 5). 

**Figure 6:**
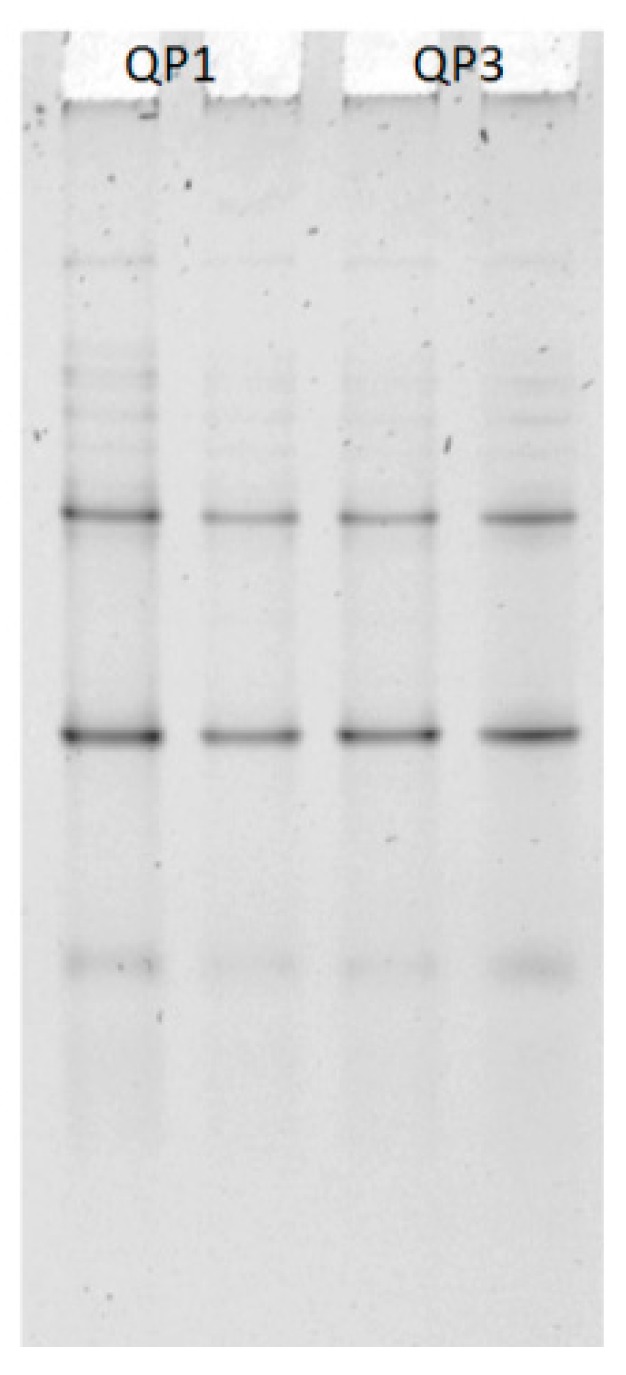
PCR-TTGE analysis of V3 16S rRNA fragments from Poro cheese samples, from 2 productions QP1 and QP3.

It is clear that cheeses from QP1 have higher values in every case, QP3 cheeses factory is a smaller and familial. Concerning the presence of fecal contamination bacteria, no coliforms were found in Poro cheese samples. This showed a good sanitary quality of those products using raw milk. DNA extracts were obtained from cheeses and after PCR amplification, were migrated on a TTGE. This exercise allowed confirming the homogeneity of Poro cheese microbiological population because the cheeses presented the same finger prints with two major and five minor bands (Figure 6). 

After numeration, 112 isolates were randomly picked from plates containing less than 300 colonies. The isolates were first characterized by conventional microbiology methods. The genera *Enterococcus* and *Lactobacillus* were identified with the species *L. plantarum, L. pentosus, L. farciminis and L. rhamnosus.* By the molecular approach, *Lactobacillus* and *Enterococcus* were identified. 

The principal species identified (Table 2) has been identified previously in other cheese around the world [14,17,44,45]; *L. plantarum, L. rhamnosus* and *L. pentosus* were found at least in two other Mexican cheeses [46,47], while *L. brevis* has been identified only in Poro chees (Table 5). 

## 4. Conclusions

This study has established the range of physicochemical, textural, rheological, color, sensorial, and microbiological attributesof Poro cheese variables. Cheese producers and a trained panel evaluated the overall acceptability of Poro cheese by attributes, allowing the construction of an external preference map for its overall acceptability. The panel generated fifteen descriptive attributes to characterize the appearance, aroma, flavor and texture of Poro cheese. High scores for flavor descriptors were related to a greater acceptability and high scores for elasticity, humidity and layers resolution descriptors were related to a lower acceptability. The results of variance analysis and means comparison showed differences among cheese samples for eight of the fifteen attributes. Despite the differences found in physicochemical variables and the marked preference of producers for QP4, no significant difference was found between the overall acceptability of the cheeses evaluated by habitual consumers. Artisanal cheese has additional characteristics that are attributed to the presence of microorganisms. In this research the main Poro cheese LAB was identified as *L. plantarum*, which is most frequently found lactic acid bacteria. The obtained resultscomprising parameters will help Poro cheese to support its origin appellation process request.

## Figures and Tables

**Table 1:** Sensory descriptors, definitions and references for the evaluation of Poro cheese.

Descriptor	Definition	Reference and Intensity
Color	Color from white to yellow	White adhesive paper (Janel) YI (−19.18), I = 1.5 Yellow adhesive paper (Post-it) YI (53.63), I = 11
Layers resolution	Space resolution due to the layers arrangement during cheese pressing	Craker Habanera (Gamesa), I = 5 Banderilla puff pastry (El globo), I = 11.5
Sour milk aroma	Aromas associated with the fermented sour milk	Lactic acid, 0.05% in milk, I = 4.5 Lactic acid, 1% in milk, I = 9
Butter aroma	Aromas associated with the butter	Diacetyl, 5 drops in 100 mL of milk, I = 4 Diacetyl, 10 drops in 100 mL of milk, I = 9
Propionic acid aroma	Strong, irritating smell associated with mature cheese	Propionic acid 5 drops in 100 mL of milk, I = 5 Propionic acid 15 drops in 100 mL of milk, I = 10
Tactile hardness	Force required to compress a sample piece between the fingers	Cocktail sausage (Swan), I = 4.5 Cut into cubes of 6 mm by 6 mm raw carrot, I = 13
Tactile creaminess	Sensation associated with the fat present in the sample at the time of touching	Cotija cheese (Esmeralda, Distribuidora de LácteosAlgil), I = 4 Philadelphia Cheese (Kraft Foods Inc.), I = 13
Sandiness	Sensation associated with small particles of sand	Panela cheese (LALA), I = 5 Parmesan cheese (Kraft Foods Inc.), I = 12
Elasticity	Sample capacity to recover its original shape when compressed with the molars	Cotija cheese (Esmeralda, Distribuidora de LácteosAlgil), I = 4 Panela cheese (LALA), I = 10
Humidity	Amount of water perceived in the oral cavity	Cotija cheese (Esmeralda, Distribuidora de LácteosAlgil), I = 4 Panela cheese (LALA), I = 11
Hardness	Force required to penetrate the simple with molars	Cocktail sausage (Swan), I = 3 Cut into cubes of 6 mm by 6 mm raw carrot, I = 11
Saltiness	Basic taste sensation occasioned by salts	Sodium chloride, 0.35% in water, I = 6 Sodium chloride, 0.5% in water, I = 11
Sourness	Basic taste sensation occasioned by acids	Citric acid, 0.2% in water, I = 5 Citric acid, 0.5% in water, I = 11
Global taste intensity	Force of cheese flavor	Cotija cheese (Esmeralda, Distribuidora de LácteosAlgil), I = 12.5
Global taste duration	Duration of cheese flavor	Cotija cheese (Esmeralda, Distribuidora de LácteosAlgil), I = 12

YI, Yellowing index; I, Intensity.

**Table 2:** Medium and incubation conditions.

Group Targeted	Medium	Incubation Temperature (°C)	Incubation Time	Anaerobic or Aerobic Conditions
Total count	PCA	30	72 h	A
Total coliforms	VRBA	30	24 h	A
Molds	OGA	25	5 days	A
Yeasts	OGA	25	5 days	A
*Enterococcus* spp.	BEA	37	48 h	A
Halotolerant flora	BHI + 5% NaCl	25	48 h	A
*Lactococcus mesophilic*	M17	25	48 h	A
*Lactobacillus mesophilic*	MRS pH 5.7	25	48 h	An
*Lactobacillus thermophilic*	MRS pH 5.7	42	48 h	An

A, aerobic; An, anaerobic conditions; PCA, plate count agar; VRBA, Violet red bile agar; OGA, Oxitetracycline glucose agar; BEA, Bile esculin agar; BHI, Brain heart infusion agar; M17 agar; MRS, Man, Rogosa and Sharpe agar (Biokar, Beauvais, France).

**Table 3:** Chemical composition of Poro cheese.

Factory Code	Chemical Composition							Mean Texture Profile Analyses (TPA)					Rheological Characteristic		Color Measurement			
	Fat (%)	Moisture (%)	Protein (%)	NaCl (%)	Ca^2+^ (%)	pH	a_w_	Hardness (N)	Adhesiveness (Ns)	Cohesiveness	Springiness	Chewiness (N)	Storage module G’ (kPa)	Loss modules G” (kPa)	Luminosity	YI	Chrome	Hue *
**QP1**	29.8 ^A^	34.8 ^A^	24.5 ^A^	3.9 ^A^	0.28 ^A^	4.9 ^A^	0.947 ^A,B^	11.92 ^A^	−0.000 ^A^	0.42 ^A^	0.83 ^A^	4.08 ^A^	510.69 ^A^	110.62 ^A^	86.33 ^A^	32.42 ^A^	17.37 ^A^	90.08 ^A^
**QP2**	31.3 ^B^	31.5 ^B^	39.4 ^B^	2.3 ^B,C^	0.12 ^B^	4.3 ^B^	0.953 ^A^	14.89 ^A,D^	−0.313 ^B^	0.40 ^A^	0.63 ^B^	3.77 ^A^	424.15 ^A^	117.70 ^A^	87.49 ^A,B^	36.02 ^B^	19.70 ^BC^	89.59 ^A^
**QP3**	37.0 ^C^	28.1 ^B^	26.3 ^C^	2.5 ^B,C^	0.19 ^A,B^	4.6 ^C^	0.930 ^B^	29.63 ^B^	−0.176 ^C^	0.38 ^A^	0.80 ^A^	9.01 ^B^	1081.14 ^B^	177.92 ^B^	89.06 ^B^	37.92 ^B^	21.27 ^B^	89.46 ^A,B^
**QP4**	31.2 ^B^	33.6 ^A^	27.4 ^B,C^	2.0 ^C^	0.12 ^B^	4.7 ^A,C^	0.954 ^A^	23.86 ^C^	−0.001 ^A^	0.47 ^A^	0.89 ^A^	9.97 ^B^	281.91 ^C^	67.40 ^C^	89.41 ^B^	40.00 ^B,C^	22.53 ^B^	88.91 ^B^
**QP5**	30.6 ^A,B^	37.0 ^A,C^	26.8 ^C^	2. 7 ^B^	0.15 ^B^	4.4 ^B,C^	0.955 ^A^	16.63 ^D^	−0.643 ^D^	0.41 ^A^	0.74 ^A,B^	5.10 ^A^	487.05 ^A^	117.80 ^A^	90.01 ^B^	31.89 ^D^	36.3 ^D^	61.45 ^C^

^A–D^ Different superscript letters within same column indicate significant differences (*p* ≤ 0.05); YI, Yellowing index; * Values expressed as degrees.

**Table 4:** Intensity scores for the sensory attributes identified of Poro cheese.

Descriptor	Cheeses Factory Code				
	QP1	QP2	QP3	QP4	QP5
Color	6.73 ^A^	7.42 ^A^	7.4 ^A^	6.01 ^B^	7.04 ^A^
Layers resolution	7.34 ^A^	7.12 ^A^	5.49 ^B^	5.79 ^B^	6.79 ^A^
Sour milk aroma	7.71 ^A^	8.07 ^A^	7.92 ^A^	7.80 ^A^	7.97 ^A^
Butter aroma	7.19 ^A^	6.85 ^A^	7.17 ^A^	7.06 ^A^	6.66 ^A^
Propionic acid aroma	7.81 ^A^	7.75 ^A^	8.21 ^A^	8.20 ^A^	7.77 ^A^
Tactile hardness	9.93 ^B,C^	9.92 ^B,C^	10.94 ^A^	9.52 ^C^	10.33 ^A,B^
Tactile creaminess	6.39 ^A,B^	5.34 ^C,D^	5.64 ^D^	6.85 ^A^	5.91 ^B,C^
Sandiness	7.50 ^A^	6.79 ^A^	7.13 ^A^	6.86 ^A^	7.51 ^A^
Elasticity	5.61 ^A^	5.60 ^A^	5.25 ^A^	5.51 ^A^	5.56 ^A^
Humidity	4.71 ^A^	4.27 ^A,B^	3.73 ^C^	4.46 ^A^	3.92 ^B,C^
Hardness	6.84 ^A,B^	7.11 ^A^	6.96 ^A^	6.12 ^B^	7.27 ^A^
Saltiness	7.76 ^A^	7.77 ^A^	7.37 ^A^	7.14 ^A^	7.18 ^A^
Sourness	7.45 ^C^	8.39 ^A^	7.98 ^A,B^	7.75 ^B,C^	7.79 ^B,C^
Global taste intensity	9.62 ^A^	9.65 ^A^	9.20 ^A,B^	8.88 ^B,C^	8.68 ^C^
Global taste duration	9.02 ^A^	8.88 ^A^	8.72 ^A^	8.62 ^A^	7.9 ^B^

^A–D^ Different superscript letters within same row indicate significant differences (*p* ≤ 0.05).

**Table 5:** Main identified lactic acid bacteria of Poro cheese.

Poro Cheese				Other Mexican Cheeses		
Accession No.	% Identity	Closed Relative	LAB Population %	Chihuahua ^A^	Doble Crema ^B^	Cotija ^B^
HQ449670.1	99	*Lactobacillus fermentum*	15	+		
AB300210.1	100	*Lactobacillus plantarum*	52	+	+	
AB289102.1	99	*Lactobacillus farciminis*	11		+	
JN415185.1	100	*Lactobacillus rhamnosus*	11	+	+	
FR871760.1	99	*Lactobacillus pentosus*	4		+	+
AB548882.1	99	*Lactobacillus brevis*	4			
GU122150.1	100	*Enterococcus faecium*	4	+	+	

^A^ Renye et al. 2011 [46], ^B^ Morales et al. 2011 [47].
